# Optical Imaging-Based Guidance of Viral Microinjections and Insertion of a Laminar Electrophysiology Probe Into a Predetermined Barrel in Mouse Area S1BF

**DOI:** 10.3389/fncir.2021.541676

**Published:** 2021-05-13

**Authors:** Victor M. Mocanu, Amir Shmuel

**Affiliations:** ^1^McConnell Brain Imaging Centre, Montreal Neurological Institute, McGill University, Montreal, QC, Canada; ^2^Department of Neurology and Neurosurgery, McGill University, Montreal, QC, Canada; ^3^Department of Physiology, McGill University, Montreal, QC, Canada; ^4^Department of Biomedical Engineering, McGill University, Montreal, QC, Canada

**Keywords:** functional localization, optical imaging of intrinsic signals, viral microinjection, optogenetics, neurophysiology, laminar probe, cortical columns, barrel field

## Abstract

Wide-field Optical Imaging of Intrinsic Signals (OI-IS; Grinvald et al., 1986) is a method for imaging functional brain hemodynamic responses, mainly used to image activity from the surface of the cerebral cortex. It localizes small functional modules – such as cortical columns – with great spatial resolution and spatial specificity relative to the site of increases in neuronal activity. OI-IS is capable of imaging responses either through an intact or thinned skull or following a craniotomy. Therefore, it is minimally invasive, which makes it ideal for survival experiments. Here we describe OI-IS-based methods for guiding microinjections of optogenetics viral vectors in proximity to small functional modules (S1 barrels) of the cerebral cortex and for guiding the insertion of electrodes for electrophysiological recording into such modules. We validate our proposed methods by tissue processing of the cerebral barrel field area, revealing the track of the electrode in a predetermined barrel. In addition, we demonstrate the use of optical imaging to visualize the spatial extent of the optogenetics photostimulation, making it possible to estimate one of the two variables that conjointly determine which region of the brain is stimulated. Lastly, we demonstrate the use of OI-IS at high-magnification for imaging the upper recording contacts of a laminar probe, making it possible to estimate the insertion depth of all contacts relative to the surface of the cortex. These methods support the precise positioning of microinjections and recording electrodes, thus overcoming the variability in the spatial position of fine-scale functional modules.

## Introduction

Optogenetics activates light-sensitive ion channels – or pumps – termed opsins at a physiologically relevant, millisecond-scale on/off kinetics (Zhang et al., 2010; Fenno et al., 2011; Yizhar et al., 2011). Depending on whether they allow cations to cross down their gradient or they pump anions or protons across the cell membrane, opsins can activate (Boyden et al., 2005; Zhang et al., 2008; Gunaydin et al., 2010; Volkov et al., 2017) or inactivate (Zhang et al., 2007; Gradinaru et al., 2008, 2010), respectively, specific populations of neurons. For a wide range of experimental objectives and hypothesis, optogenetics can be combined with readout techniques to measure *in vivo* neural activity, such as extracellular electrophysiology recordings (Gradinaru et al., 2007; Scanziani and Hausser, 2009; Dugué et al., 2012; Yang et al., 2017, 2018), functional imaging techniques such as functional magnetic resonance imaging (fMRI) (Lee et al., 2010) or intrinsic optical imaging (Scott and Murphy, 2012; Chernov et al., 2018), and behavioral observations (Adamantidis et al., 2007; Gradinaru et al., 2007; Han et al., 2017). To study cortical processing at the scale of cortical columns, it is important to optimize the opsin gene introduction into neurons around a predetermined, small cortical module and the readout from such a module.

Selecting the cortical sites for microinjections and for inserting the recording electrodes is commonly done by using stereotaxic coordinates referenced from structural brain atlases (Kirkcaldie et al., 2012; Watson et al., 2012; Knutsen et al., 2016; Yang et al., 2017; Paxinos and Franklin, 2019). This approach has been commonly used and optimized for applying optogenetics in rodents (Cetin et al., 2007). However, atlas-based positioning of microinjections and electrodes provides only an approximation of the true locations of functional modules, as there can be significant inter-individual (between-subject) variation (Jellema et al., 2004; Oberlaender et al., 2012; Knutsen et al., 2016; Paxinos and Watson, 2017). This is especially problematic for small functional modules such as cortical columns with diameters as small as 200 – 300 microns. For example, maps of cortical columns for the same functional feature from the same cortical area in two different individuals may feature two different organizations: a radial pinwheel organization (Bonhoeffer and Grinvald, 1993) or a linear organization (Shmuel and Grinvald, 2000). Therefore, localizing an insertion with high precision with respect to cortical columns cannot be based solely on stereotactic coordinates.

A different method used for guiding the insertion of an electrode prior to recording from a functional module is based on multiple insertions of an electrode to sparsely sample the responses from the region of interest. Previous studies located barrels in the rodent primary somatosensory barrel field (S1BF) by systematically inserting electrodes in a trial-and-error approach while administering a stimulus to characterize the cortical column properties and performing *post hoc* histology to validate the insertion site (Andermann and Moore, 2006; Yang et al., 2017; Laboy-Juárez et al., 2019). However, this method takes a long time to perform, it can damage the cortex before the experiment has even begun, and it gives only a partial view and sparse sampling of a small area of barrels and septa.

The purpose of the method we present here is to enable high-precision targeting of injections and neurophysiological recordings relative to small functional modules in rodents. To this end, we have devised a protocol to guide microinjections and electrode insertions more efficiently and more precisely than the methods described above. Based on stimulus-evoked hemodynamic responses imaged with Optical Imaging of Intrinsic Signals (OI-IS) (Grinvald et al., 1986), the protocol allows guiding the insertions of microinjection pipettes and/or recording electrodes around or into small functional modules.

Optical Imaging of Intrinsic Signal primarily measures the local changes in the content of deoxy-hemoglobin (deoxy-Hb), oxy-Hb, and the total volume of Hb elicited by neural activation (Grinvald et al., 1986; Frostig et al., 1990; Grinvald et al., 1999; Berwick et al., 2008). These changes cause changes in the absorption of light of specific wavelengths shone onto the surface of the cortex. As we will demonstrate, the results can be used for several steps in an optogenetics experiment. They can be used for guiding viral microinjections around a small target area as was previously demonstrated in monkey area V1 (Ruiz et al., 2013), optimizing the photostimulation used for optogenetics as described by Yizhar et al. (2011), and guiding electrophysiology electrode to a functional module as small as a single barrel with a diameter of 200 microns. Regardless of the brain region to investigate, the principle is the same: apply stimuli known to activate the functional module and obtain a spatially mapped stimulus-activated hemodynamic response. The response amplitude needs to be sufficient to create visible spatial contrast between modules that respond preferentially to the specific stimulus and other modules in the area. Throughout the text, we will use the terms ‘targeted module,’ ‘pre-defined module,’ or ‘targeted barrel’ to refer to the small stimulus-activated region around which we aim to perform microinjections or into which we guide the electrode insertion.

Optical Imaging of Intrinsic Signals resolves fine-scale modules showing hemodynamic responses that correlate with neuronal responses (Grinvald et al., 1986; Shmuel and Grinvald, 1996). The imaging can be performed with a low degree of invasiveness – through the intact (in mice) or thinned skull (in mice and rats), which is optimal for survival experiments.

Optical Imaging of Intrinsic Signals-based guidance of electrode insertions to small functional modules was previously introduced in large animals (Grinvald et al., 1986; Shmuel and Grinvald, 1996; Arieli and Grinvald, 2002; Shmuel et al., 2005). In rats, OI-IS can localize individual cortical columns and barrels in area S1 (Drew and Feldman, 2009; Bortel et al., 2019). OI-IS has recently gained ground as a means of localizing cortical targets for optogenetics manipulation and investigation (Ruiz et al., 2013; Chernov et al., 2018; Yang et al., 2018). This targeting functionality using OI-IS resembles previous studies that localized cortical functional columns in non-human primates, with the purpose of recording from them (Ts’o et al., 2001; Shmuel et al., 2005; Chen et al., 2008; Lu et al., 2010; Tanigawa et al., 2010). However, only one article has described the OI-IS as explicitly tailored and designed to guide optogenetics viral microinjections (Yang et al., 2018). Our paper presents detailed methods for OI-IS-based guidance of optogenetics viral microinjections close to – and around a predetermined small functional module and extends the OI-IS-based guidance to the readout/recording from within such a module.

Our current study focuses on the guidance of microinjections of viral vectors around a small functional module in the rodent cortex and – following an incubation period – the guidance of an electrode insertion into a pre-defined module for electrophysiology recordings. These methods also allow the user to visualize the spatial spread of the optogenetics photostimulation and – at higher magnification – to estimate the cortical depth of the electrode contacts by imaging the upper recording contacts visible outside of the cortex (Sotero et al., 2015). We verify that the method indeed results in the insertion of the electrode into the targeted module by visualizing the insertion site in images of the histology-processed tissue. Overall, the methods we describe allow for precise and consistent functional localization of small cortical structures with a minimal degree of invasiveness as required for optogenetics experiments.

## Materials and Methods

### (1) Pre-surgery Preparation

All procedures were approved by the animal care committees of the Montreal Neurological Institute and McGill University and were carried out in accordance with the guidelines of the Canadian Council on Animal Care. Adult C57BL/6 10–15 weeks old female and male mice were used for all experiments. The choice of mice, and their genotype and phenotype must be made judiciously according to the specific experimental needs.

A list of equipment items and materials commonly used in the experiments we describe is provided in Table 1. Before experiments, sterilize surgical instruments using a hot bead sterilizer (Germinator 500, Stoelting, IL, United States) or by autoclaving. Apply aseptic protocols to the surgery and recovery areas.

**TABLE 1:** Materials.

Name of material/Equipment	Company	Catalog number	Comments/Description
0.9% Saline Sodium Chloride Injection Bag	Baxter Healthcare Corporation	288-0006AA	SURGERY. To safely flush tissue other than the brain
Hanks’ Balanced Salt solution (HBSS)	MilliporeSigma Canada Co.	55021C	SURGERY. Solution applied topically to the brain during surgery, to keep the brain from drying
Sugi cellulose absorbent triangles	Kettenbach GmbH & Co. KG, Germany	001911	SURGERY. To absorb excess solutions or blood from tissues
Dowsil Silicone sealant	Dow Corning	3140 90ML MIL-A-46146	SURGERY. For a rigid hydrophobic silicone chamber for holding HBSS
Ethilon Sutures 5-0	Ethicon-Johnson & Johnson	661H (nylon monofilament, FS-2, 45 cm strands)	RECOVERY SURGERY. To connect the skin flaps after the recovery surgery
Polysporin Complete ointment	Johnson & Johnson	60245-43775	RECOVERY SURGERY. To avoid infections during post-op recovery period; it contains three antibiotics plus lidocaine hydrochloride
Syringe Priming Kit	Chromatographic Specialties Inc.	HPRMKIT	VIRAL MICROINJECTIONS. Indispensable to load up the virus into the syringe
Syringe 10 μL removable needle	Hamilton	701RN	VIRAL MICROINJECTIONS. To hold the virus
Glass micropipettes tubes	World Precision Instruments Inc.	18100-3 (3IN BOROSIL GL 1.0 MM OD)	VIRAL MICROINJECTIONS. They will need to be pulled into shape
PHD ULTRA^TM^ Syringe Infuse/Withdraw Programmable Micropump	Harvard Apparatus	70-3007	VIRAL MICROINJECTIONS. For pumping the viral solution through the micropipette at an optimal low rate
Master9 Programmable Pulse Stimulator	A.M.P.I.		STIMULATION. For timing the sensory stimuli
Electrical constant current stimulator	World Precision Instruments Inc.	A365	STIMULATION. For generating electrical current sent to the piezoelectric actuator
Piezoelectric actuator	Piezo Systems Inc.	PSI-5A4E, Y-poled, double quick-mount bender	STIMULATION. For whisker stimulation
VDAQ 3001	Optical Imaging Inc.		OPTICAL IMAGING. For data acquisition
Camera lens	Nikon	1987 (60 MM F/2.8 D-AF)	OPTICAL IMAGING. Optimal for wide-field OI-IS
Camera zoom lens	Edmund Optics	VZM1000i	OPTICAL IMAGING. Optimal for imaging at high-magnification
LED 530 nm	Mightex	BLS-LCS-0530-15-22	OPTICAL IMAGING. LED for Optical Imaging
Electrode for acute insertion and recordings	NeuroNexus	A1 × 32-50-177-A32	NEUROPHYSIOLOGY. Electrode characteristics customizable to the experimental needs
Optogenetic virus	Neurophotonics Centre – Molecular Tools Platform – Ulaval	Viral vector selected according to the scientific question	OPTOGENETICS. The viral vector to be injected into the brain
LED 470 or 595 nm	ThorLabs	M470F3, M595F2	OPTOGENETICS. LED for optogenetic photostimulation
Multimode optical fiber	ThorLabs	FPC-1000-37-02SMA	OPTOGENETICS. Multimode Fiber Patchcord with 0.37 NA, 1000 μm core diameter and SMA connectors
DiI Vybrant cell-labeling solution	Life Technologies	V22885	HISTOLOGY. For marking the track of the electrode
Cytochrome C from bovine heart	Sigma Aldrich	C3131-10MG	HISTOLOGY. For cytochrome oxidase staining
DAB (3,3′-Diaminobenzidine tetrahydrochloride)	Sigma Aldrich	D5905	HISTOLOGY. For cytochrome oxidase staining
DAPI (4′,6-Diamidino-2-Phenylindole, Dilactate)	Thermo Fisher Scientific	D3571	HISTOLOGY. For DAPI fluorescence counterstaining

(1.1) Induce and then systematically maintain an appropriate plane of anesthesia and analgesia for the surgical procedure. We use ‘Mouse Cocktail’ combination of ketamine 80–100 mg/kg, xylazine 10 mg/kg and acepromazine 2.5–3 mg/kg, injected I.P. to induce a surgical plane of anesthesia, followed by ketamine 80–100 mg/kg and xylazine 10 mg/kg to maintain anesthesia (Flecknell, 2009). For analgesia, we inject an initial one-time bolus of carprofen 5–10 mg/kg subcutaneously (Flecknell, 2009; Ferry et al., 2014). To verify the surgical level of anesthesia, check for the absence of whisking and withdrawal reflex during a hindpaw painful pinch, and the absence of blinking upon eye contact (to be done while also constantly hydrating the cornea with a protective ophthalmic ointment). In addition, monitor the heartbeat, and make sure the respiration is regular with no signs of gasping (Flecknell, 2009; Ferry et al., 2014).

The anesthetics used should maintain neurophysiological activity and neurovascular coupling as much as possible unchanged. For this, a light plane of anesthesia during the recording sessions must be kept constant by systematically monitoring the vital signs and reflexes, as well as the electrophysiology readout (Flecknell, 2009). Any systematic increases in the heart beat or respiration rate must be counteracted by additional low doses of injectable anesthetic. Conversely, if the vital measures decrease and the spontaneous electrophysiological activity is visibly poor, provide the appropriate antagonist (Flecknell, 2009; Ferry et al., 2014).

(1.2) If using a piezoelectric whisker stimulation, tape or cut away all the same-side whiskers that will not be stimulated during the experiment. Use the surgical microscope to identify these whiskers and ensure their cutting.
(1.3) Position the animal in a small-animal stereotaxic frame (David Kopf Instruments, CA, United States) in a manner consistent with the conventions of the reference atlas, and provide free-flowing oxygen via a nose cone (Ferry et al., 2014). During electrophysiology recordings, switch to a mixture of 70% medical air and 30% oxygen. To reduce discomfort, use non-penetrating ear bars, covered with a drop of Xylocaine ointment (Aspen Pharmacare Canada Inc., ON, Canada).

### (2) Stereotaxic Surgery and Skull Thinning or Craniotomy

(2.1) Cut the skin longitudinally along the midline with a scalpel and retract it laterally with a clamp. Remove soft tissue and dry off the exposed skull surface using cotton swabs. Administer topical epinephrine 1 mg/mL (Epiclor, McCarthy & Sons Service, AB, Canada) sparingly or sterile isotonic 0.9% NaCl saline in case of muscle or bone bleeding.
(2.2) Flush the surgical site with small amounts of topical lidocaine hydrochloride 2% (Wyeth, NJ, United States). As soon as the bone has been pierced, do not use lidocaine nor epinephrine, as they will modify the animal’s physiology and brain state. Instead, use sterile isotonic 0.9% NaCl saline (Baxter Healthcare Corporation, IL, United States) or preferably Hanks’ Balanced Salt solution (HBSS) (MilliporeSigma Canada Co., ON, Canada) to thoroughly clean the surgical site. Because bleeding can impact the quality of the OI-IS, any sources of bleeding must be controlled immediately using persistent flushing with HBSS and absorbing the mixture of blood and HBSS with cotton swabs or Sugi cellulose absorbent triangles (Kettenbach GmbH & Co. KG, Germany) without ever touching the actual brain surface or the dura mater.
(2.3) Find on the skull the bregma, the rostrocaudal and mediolateral coordinates for the cortical region of interest (Ferry et al., 2014; Paxinos and Franklin, 2019).
(2.4) Drill the cranium with a fine micro-drill tip (Fine Science Tools, BC, Canada) under the microscope, using low-force long movements. We observed that constantly applying sterile saline or HBSS to the bone before drilling makes it soft and spongy, and smoothens the drilling process.
(2.4.1) For a survival microinjection experiment, thin the bone until it is flexible under gentle pressure. Homogenize and polish the surface with a silicone polisher micro-drill tip. The bone will be made transparent via an HBSS or silicone oil-filled silicone chamber in step 2.5 (Arieli and Grinvald, 2002).
(2.4.2) For an acute electrode insertion, perform a craniotomy by carefully delineating an area of ∼3 millimeter (mm) × 3 mm, and thinning the perimeter of this area until it can be safely pierced. Then gently lift the central piece of bone, while avoiding damage to the brain. For electrophysiology recordings, place a stainless steel skull screw in a region of no-interest in the contralateral hemisphere to use as a ground and reference.
(2.5) Around either the thinned or removed part of the bone, lay down in successive layers a thin-walled silicone chamber (Dow Corning, MI, United States). Allow it to harden, then fill it with HBSS. Make sure the silicone does not spill into the thinned bone nor into the craniotomy, by applying it in several small layers that build upon each other, before it hardens solid.

### (3) Stimulation

**FIGURE 1:**
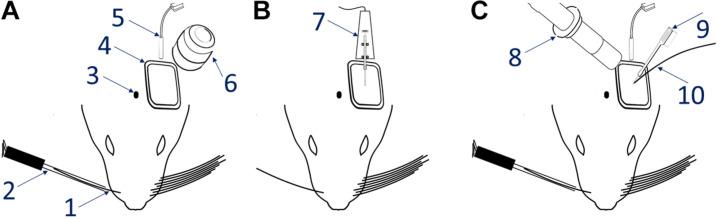
Experimental setup. The left-most panel **(A)** shows the OI-IS recommended setup, before either microinjections or electrophysiology recordings. The middle panel **(B)** shows the optogenetics virus microinjection setup using a micropump directly connected to a mounted 10 microliter syringe. The right-hand panel **(C)** is the setup for acute electrophysiology recordings together with optogenetics photostimulation. 1 – Single whisker contralateral to imaging/recording site, 2 – Piezoelectric device with whisker adapter, 3 – Bregma marking on the skull, 4 – Cortical chamber with silicone chamber, 5 – 530 nm Optical Imaging illumination, 6 – Optical Imaging lens, 7 – Microinjector pump and syringe, 8 – Zoom lens imaging of the insertion site, 9 – Multichannel electrode, 10 – Optogenetics illumination and optic fiber.

(3.1) Set up the hardware, as required. Configure the sensory stimulation and OI-IS setups as shown in Figure 1A.

(3.1.1) Turn on the stimulation system. In our setup, we use a constant current stimulus isolator (World Precision Instruments, FL, United States) to deliver bipolar impulses to a 0.58 mm-thick rectangular piezoelectric double-quick-mount actuator (Mide Technology – Piezo, MA, United States), which can deflect ± 270 microns. This deflection is amplified by extending the length of the device using a 3D-printed hollow plastic micropipette (Armstrong-James et al., 1992; Welker et al., 1993), although even a 200 micron deflection should be sufficient to elicit cortical responses (Welker et al., 1993). When the stimulus isolator delivers pulses of 400 microamperes, the 3D-printed micropipette will be displaced at a speed of approximately 35 microns per millisecond, optimal for eliciting cortical evoked responses (unpublished observations).
(3.1.2) Turn on the impulse generator. In our setup, we use a Master-9 Programmable Pulse Stimulator [A.M.P.I., Israel] to deliver 245 milliseconds long square-wave pulses at 4 Hz to the piezoelectric actuator.
(3.2) Prepare the somatosensory stimulation: insert each individual whisker inside the micropipette attached to the piezoelectric device, which is deflected with a ramp-hold-return paradigm at a frequency close to the rodent natural whisking range (Mitchinson et al., 2011; Clancy et al., 2015; Knutsen et al., 2016; Laboy-Juárez et al., 2019). The micropipette should ideally reach as close as 2 mm from the face, and deflect only rostro-caudally, a preferred direction for the whisker sensory system (Andermann and Moore, 2006; Jacob et al., 2008; Le Cam et al., 2011; Vilarchao et al., 2018). Ideally, different micropipettes should be moved without touching any of the other micropipettes or intact whiskers.

### (4) Optical Imaging of Intrinsic Signals

**FIGURE 2:**
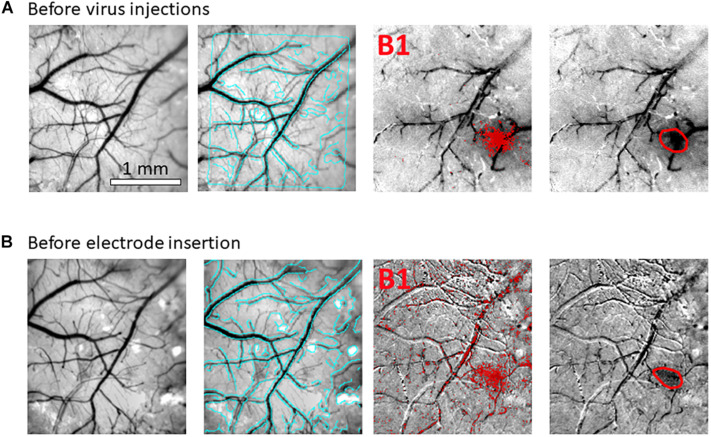
Hemodynamic responses to single-whisker piezoelectric stimulations. **(A)** OI-IS through the thinned skull for guiding microinjections. The 2 left-most panels shows an image of the surface of cortex, taken under green light illumination (peak wavelength of 530 nm) at the beginning of the experiment to obtain the pial vessel topography. The cyan curves are edges of the pial vessels computed before the insertion of the electrode (second panel in **B**) and superimposed on the pial vessels image before the injections, for demonstrating the alignment of the imaged regions and responses. The third panel presents the hemodynamic response to single whisker (whisker B1) stimulation, averaged over 10 trials. Pixels with superimposed red dots showed statistically significant responses. The fourth column shows the perimeter encompassing the responding region, obtained by computing the convex hull around all clusters of connected responding pixels that showed statistically significant response. **(B)** OI-IS following a craniotomy, for guiding the insertion of the recording electrode and the positioning of the optic fiber. All four panels are identical in scope to those presented in **(A)**. The cyan colored curves present the edges of the pial vessels computed (using Canny edge detection) from the green image obtained before electrode insertion.

**FIGURE 3:**
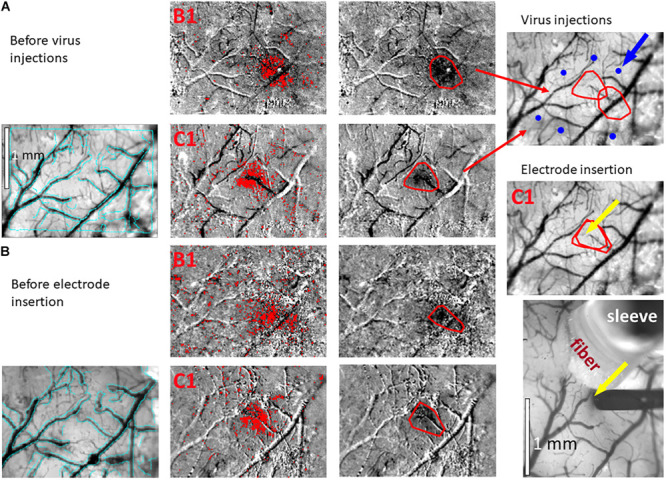
Hemodynamic responses to single-whisker piezoelectric stimulations. **(A)** OI-IS through the thinned skull for guiding microinjections. The left-most panel shows an image of a mouse’s right hemisphere. This image was taken under green light illumination (peak wavelength of 530 nm) at the beginning of the experiment to obtain the pial vessel topography. The cyan curves are edges of the pial vessels computed before the insertion of the electrode (left-most panel in **B**) and superimposed on the pial vessels image before the injections, for demonstrating the alignment of the imaged regions and responses. The scale bar represents 1 mm. Each row presents OI-IS steps in their chronological order, for stimulating a single whisker: B1 or C1, respectively. The second column presents images of hemodynamic responses to single whisker stimulation, averaged over ten trials. Pixels with superimposed red dots showed statistically significant responses. The third column shows the perimeter (red curves) encompassing the responding region, obtained by computing the convex hull around all clusters of connected responding pixels. The image in the fourth column overlays the perimeters computed in response to stimulating the B1 and C1 whiskers on top of the green reference image to localize the hemodynamic response with respect to the pial vessels topography. It outlines the overall stimulus-activated region comprising the responses to all the stimulated barrels (red curves), so that microinjections (blue circles; indicated by a blue arrow) can be planned around it, as close as possible to the barrels of interest without damaging these barrels and/or pial blood vessels. **(B)** OI-IS following a craniotomy, for guiding the insertion of the recording electrode and the positioning of the optic fiber. The first three columns are identical in scope to those presented in **(A)**. The cyan colored curves present the edges of the pial vessels computed (using Canny edge detection) from the green image obtained before electrode insertion. The upper image in the right-most column shows in red curves the delineation of the responses stimulating whisker C1 before the injections and before the insertion of the electrode. The yellow arrow points to the position of electrode insertion. The lower image is a high-magnification image of the region where the electrode was inserted. The experimenter selects a site for recording (yellow arrow) in the center of a barrel whose location is estimated by the hemodynamic response, along with the region where photostimulation will be applied. In the bottom-right panel, the electrode is shown after it was inserted at the recording site (yellow arrow). The tip of the optic fiber is placed on top of the cortical surface immediately adjacent to the electrode; the fiber’s protective outer sleeve is seen out of focus. The scale bar represents 1 mm.

**FIGURE 4:**
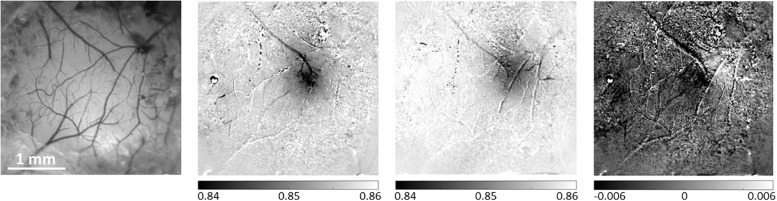
Differential analysis of evoked hemodynamic responses improves spatial contrast and definition of a single barrel activation area. The left-most panel presents a reference image taken under illumination wavelength of 530 nm, showing the pial vessel topography. The second panel shows the hemodynamic response to a single-whisker stimulation of the B1 whisker, calculated as the division of the images acquired during stimulation trials by the images acquired during control/no stimulation trials. The third panel shows a similar result for the Alpha whisker. In the third panel, the differential analysis of these two neighboring whiskers’ responses improves the spatial discrimination between the two individual but partially overlapping responses. The grayscale applied to all images represents the same range of relative responses. The scale bar represents 1 mm.

(4.1) Optical Imaging of Intrinsic Signals is performed with a monochrome Dalsa DS-21-01M60 camera fitted with a 60 mm AF Micro-Nikkor f/2.8D lens (Nikon Corporation, Japan), linked to a Brain Imager 3001M interface (Optical Imaging Ltd., Israel) and controlled by the VDAQ imaging software (Optical Imaging Ltd., Israel). Throughout all experiments, the camera resolution is 1024 × 1024 and frame rate is 30 Hz, down-sampled to a 10 Hz data frame rate. For electrophysiology insertion recordings, VZM1000i zoom lens with up to 10x-magnification (Edmund Optics, NJ, United States) in order to view and count the electrode’s upper contacts that remain above the cortical surface. This makes it possible to monitor the insertion of the probe and estimate the electrode’s cortical insertion depth.
(4.2) Turn on the 530 nanometers (nm) LED (Mightex, CA, United States), and position it such that it illuminates the entire ROI uniformly, with the peak of luminosity at the center of the region intended for microinjections (based on atlas coordinates) or insertion of a neurophysiology probe (based on the optical imaging pursued in a previous imaging session, prior to performing the microinjections). Leave it on continuously while adjusting the position of the charge-coupled device camera.
(4.3) Translate and rotate the camera until the entire ROI is within the field of view of the camera. Position the camera above the ROI, so that its optical axis is approximately orthogonal to its cortical surface. Define the imaged region within the field of view.
(4.4) Adjust the LED output to maximize the luminosity values within the area imaged, while avoiding saturation. If there are any light reflections – such as reflections caused by the silicone chamber or the HBSS inside it – keep them outside of the imaged region or try repositioning the illumination light-guide.
(4.5) Before each run, save an image of the pial vessels under green-light illumination, as a reference. The imaged ROI can be saved as a separate image, to be used in step 4.8. The topography of the cortical vessels can then be viewed *in vivo* using a surgical microscope, thus making it possible to guide the insertion of a micropipette or electrode to the small target area. It can also be used for analyzing whether the targeted module shifted for unexpected reasons.
(4.6) Use the OI-IS system to image the response to stimulating each individual whisker of interest. Experimental runs consist of ten stimulation trials (Condition 1) interleaved with ten trials of spontaneous activity (Condition 0). Each stimulation trial consists of 2 s of baseline activity, 6 s of stimulation (in our case, bidirectional whisker piezoelectric deflections), and then 2 s with no stimulus, followed by an inter-trial interval of 7 s. Optical imaging is performed throughout all stimulation and spontaneous activity trials.
(4.7) Compute a trial-by-trial single-condition map by dividing the average of images obtained during the response to the whisker (condition 1) of interest by the average of images obtained during the no-stimulus condition (condition 0; Figures 2, 3; Grinvald et al., 1986, 1999). Alternatively, or in addition, compute a trial-by-trial differential response map by dividing the average of images obtained during the response to the whisker of interest by the average of images obtained during the response to stimulating a different whisker (Figure 4; Bonhoeffer and Grinvald, 1993; Shmuel and Grinvald, 1996; Grinvald et al., 1999). Both in single condition analysis and differential analysis, we recommend subtracting the frame obtained just before the stimulation begins, to remove slow drifts in cerebral blood volume (CBV) and/or oxygenation.

For each of the trial-by-trial single-condition map and differential maps, the results obtained from the ten trials (10 stimulation blocks) within a run are used for computing the mean and standard deviation (SD), to obtain an averaged stimulus-evoked response or a difference map for the current run.

(4.8) On each of the hemodynamic response images, estimate the activated area using an automated (except for determining the statistical threshold for activation), objective algorithm, and then overlay this result on top of the ROI image from step 4.5 (Figures 2, 3). The algorithm estimates the pixel-wise mean and SD of the relative response over stimulation blocks in one or more runs. We perform pixel-wise statistical testing of the null hypothesis that there is no difference between the mean response in the stimulation condition compared to the no stimulation condition; *t*-test, *p* < 0.01. This results in a binary map of pixels where the null hypothesis was accepted or rejected. We then mask out pixels located within pial vessels segmented from the OI image taken under illumination wavelength centered at 530 nm. To eliminate spurious response-like results from single pixels, we perform pixel by pixel neighborhood connectivity analysis on the binary map from which blood vessels regions were excluded, and eliminate all responses that form clusters of 7 or less ‘connected’ pixels. ‘A connected pixel’ is defined as any pixel adjacent to the currently analyzed pixel by sharing and edge or a corner (eight pixels neighborhood). Lastly, we compute the convex hull of the remaining clustered pixels in the binary image.
(4.9) In case you stimulate whiskers individually in separate runs, repeat steps 3.2, and 4.5 – 4.8 for each whisker. In the end, superimpose a delineation of the responses of all whiskers of interest for a comprehensive overview of all the responses on the reference image of the pial vessels obtained under green light illumination.

### (5) Microinjection of Optogenetics Viral Vector

Configure the viral stereotaxic microinjection setup as shown in Figure 1B. NOTE: Set up the microinjection apparatus and surgical area in accordance with your local government and university regulations, and following previous publications on the subject (Cetin et al., 2007; Lowery and Majewska, 2010; Zhang et al., 2010; Thompson and Towne, 2018; Yang et al., 2018). We use a 10 microliter (μL) 701-RN glass micro-syringe (Hamilton, NV, United States) controlled by a PHD ULTRA programmable microinjection pump (Harvard Apparatus, MA, United States). We use a Syringe Priming Kit (Chromatographic Specialties Inc., ON, Canada) to load up just over 10 microliters of mineral oil (Millipore-Sigma Canada Co., ON, Canada).

To minimize cortical damage, use borosilicate glass micropipettes (World Precision Instruments, FL, United States) pulled to an outer diameter of the tip of 30–100 microns or smaller. If these are not available in your lab, use 36 G or higher G needles (NanoFil, World Precision Instruments, FL, United States). Beveled needles and micropipettes will penetrate the dura easier, whereas blunt ones will expel a more controlled drop of viral solution. Keep in mind that the smaller the tip’s inner diameter is, the higher the chance of tissue backflow clogging it. To prevent this, apply a constant slightly positive pressure when moving the micropipette up or down through the cortex.

(5.1) Load up the virus into the glass micropipette at a rate of 50–250 nanoliter (nL) per minute, using a piece of sterile parafilm (Yang et al., 2018) or metal foil, which is non-reactive with the virus. It serves as a shallow non-porous ‘dish’ in which to safely deposit the virus, so that it gets taken up by the micropipette. Do not allow the virus to reach past the glass micropipette and into the syringe. For our optogenetics experiments, we used the virus AAV2/8-CAG-flex-ChR2-tdTomato-WPRE with a titer of 1.5e13 genome copies per ml, as prepared by the Neuro-Photonics Centre’s Molecular Tools Platform (Université Laval, QC, Canada).

NOTE: Translation of a floxed or double-floxed inverted open-reading-frame viral genome depends exclusively on the spatially specific presence of the Cre-recombinase in Cre knock-in mice, allowing virtually 100% tropism for the targeted tissue/layer/cells (Cardin et al., 2009; Sohal et al., 2009; Zhang et al., 2010; Fenno et al., 2011).

**FIGURE 5:**
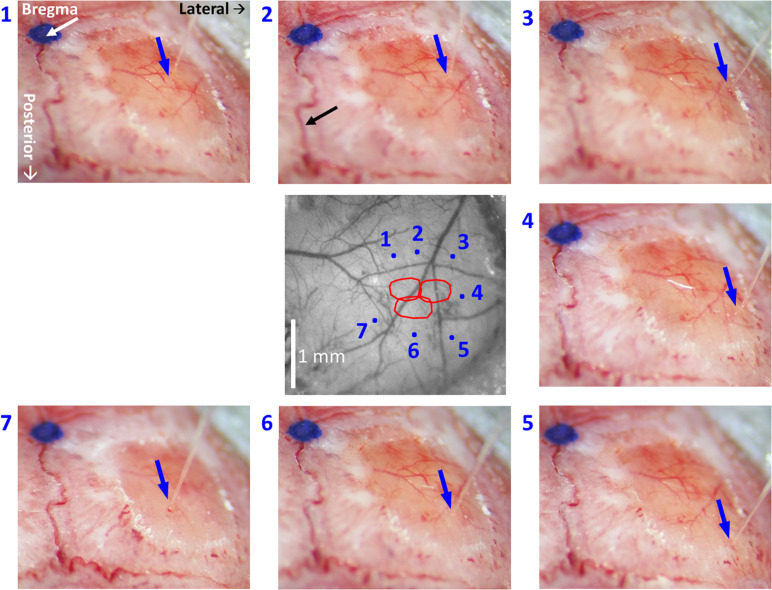
High-resolution images of each individual microinjection location, compared to the corresponding planned site. The image at the center was taken under green light illumination (peak wavelength of 530 nm). It shows the region of interest comprising all the stimulated barrels (red contours, calculated as in Figure 2, 3). Each of the seven images around it was taken after the insertion of the glass micropipette at one of the sites selected for viral micro-injection. The insertion points are indicated by blue arrows. The numbers next to the images of the micropipettes correspond to the microinjection sites displayed in the center image by blue dots. The white arrow in the top-left panel points at a blue dot: this is a fiducial we placed onto the skull to mark the bregma. In the top-middle panel, the black arrow points to the midline suture going posteriorly. The stereotaxic axis directions are marked in the top-left panel. The scale bar represents 1 mm.

(5.2) Select sites for viral microinjections in close proximity around the modules of interest while considering the lateral spread of the virus, but strictly avoiding sites close to macroscopic blood vessels. Take note of the stereotaxic location of the selected sites relative to bregma. Importantly, the site selected for microinjection should be projected onto the image of the cortical surface and pial vessels (Figures 3, 5). This will make it possible to guide the insertion of the micropipette to the selected site, while viewing the pial vessels using a surgical microscope.

(5.3) For each site, gently pierce the thinned cranium, creating a small hole with a fine needle or scalpel, while flushing thoroughly with HBSS.
(5.4) Position the glass micropipette above the insertion site, insert and lower it down to the desired cortical depth, while keeping positive pressure in it throughout the insertion to avoid clogging. The cortical depth of the insertion can be estimated relative to the point in which the micropipette first touched the surface of the cortex, based on continuous imaging of the insertion site using the OI system. Wait up to 5 min following the insertion of the micropipette, to allow the brain tissue to settle. Taking images of all micropipette insertions is highly recommended, as it is part of the documentation of the experiment.
(5.5) Inject 100–150 nL of virus solution per site, at a rate of 20–100 nL per minute. Given the original titer, this provides 1.5–2.25e12 total genome copies per microinjection. While the viral spread and infection efficiency are also significant factors in opsin expression, it has been previously reported that at least 1e12 genome copies are sufficient for a cortical transduction volume of 1 mm^3^ (Zhang et al., 2010). Note that other researchers have used as low as 6e7 viral particles per injection, with excellent results (Yang et al., 2017).

Wait 10 minutes for the injected solution to diffuse out into the tissue.

(5.6) Retract the glass micropipette up while keeping a positive pressure inside.
(5.7) Repeat steps 5.1–5.5 at other selected insertion sites.
(5.8) After completing all the microinjections for an animal, clean the glass micropipette tip with sterile isotonic saline or HBSS drips. At the end of the microinjection session, drop the micropipette with any remaining virus in a solution of 0.5% sodium hypochlorite for at least 15 min, and then dispose it in a biohazard sharps container. Disinfect the surgical area and tools with a bleach- or peroxide-based solution, not an alcohol-based one.
(5.9) Animal Recovery

(5.9.1) Clean the treated area with sterile isotonic saline or HBSS. Close the skin flaps and suture them together using absorbable sutures of size 5-0 or 6-0 (Ethicon Inc., NJ, United States).

(5.9.2) Apply Polysporin triple antibiotic cream with lidocaine topically (Johnson & Johnson Inc., NJ, United States) onto the sutured flaps. Administer isotonic sterile saline or dextrose solution subcutaneously in the back of the animal, to prevent dehydration.

(5.9.3) Monitor the animal until it recovers from anesthesia and demonstrates full mobility. If no signs of discomfort are visible, return the animal to its cage.

(5.9.4) Monitor daily and inject an analgesic agent (carprofen 5−10 mg/kg subcutaneous) for 3 days postoperatively.

### (6) Electrophysiology Recordings

(6.1) Allow 3–6 weeks (Zhang et al., 2010; Miyashita et al., 2013) for the virus to incubate and express.
(6.2) Repeat steps 1.1–4.9 of the protocol.
(6.3) Select a site(s) for electrophysiology recording. The position is guided by the OI-IS responses obtained in the session that preceded the microinjections. We perform optical imaging in preparation for the neurophysiological recordings too, both for verification and evaluation of the functionality of the modules of interest following the virus microinjections. Strictly avoid electrode insertions close to macroscopic blood vessels. The images of the responses from the two sessions can be spatially registered by aligning the pial vessel images obtained in the two sessions (Figures 2, 3). Similarly to the guidance of the insertions for microinjections, electrode insertions are also guided according to the image of the pial vessels (Figure 3, bottom-right panel).
(6.4) Configure the setup for optogenetics photostimulation together with electrophysiology recordings, as shown in Figure 1C. If applicable, switch the OI-IS lens to a high-magnification lens, to make it possible to monitor the position of insertion and the depth of the insertion based on imaging the contacts of the probe.
(6.5) Position an acute recording electrode above the selected site, with its recording axis orthogonal to the local surface of the cortex. If using a linear/laminar probe, estimate the angle relative to the cortex from multiple viewpoints, and – if needed – modify the insertion angle for an approximate orthogonal orientation relative to the cortical manifold. For post-experiment localization of the electrode track, gently dip the recording electrode shank a few millimeters in a DiI Vybrant (Life Technologies, CA, United States) cell-labeling solution (An et al., 2012; Laboy-Juárez et al., 2019) prior to inserting it into its final position.

NOTE: Electrophysiological signals sampled at 24,414 Hz are pre-processed by a PZ5 NeuroDigitizer 128-channel preamplifier (Tucker-Davis Technologies, FL, United States), and then processed and recorded using the Synapse Suite software (Tucker-Davis Technologies, FL, United States). For mouse experiments that do not require electrolytic micro-lesions, we use A1 × 32-50-177 probes with a 50 micron thick shaft (NeuroNexus, MI, United States).

(6.6) Place an optic fiber connected to high-powered light-emitting diode immediately next to the electrode. The optic fiber should be positioned approximately 0.5 mm from the dura mater, pointing to the region of cortex where the electrode is inserted, which is expected to be infected by the previously injected virus.

NOTE: Use Dr. Karl Deisseroth’s link at: https://web.stanford.edu/group/dlab/cgi-bin/graph/chart.php for a “Brain tissue light transmission calculator” to predicted irradiance values from a given user-defined optic fiber through standard mammalian brain tissue. For example, our experiments used a multimode optic fiber with numerical aperture of 0.37 and 1 mm inner core diameter (Mightex, CA, United States), connected by SMA to a high-power, fiber-coupled, 470 nm LED (ThorLabs Inc., NJ, United States) for exciting the ChR2 opsin. For an LED light power output of 6.4 milliWatt (mW), the irradiance measured at the fiber tip is 2.03 mW/mm^2^, as verified before each experiment using a digital handheld power meter (ThorLabs Inc., NJ, United States). Then, the calculated irradiance value at a cortical depth of 100 micron is 1.43 mW/mm^2^, and at 1 mm deep, it is 0.1 mW/mm^2^.

Choose the optic fiber parameters based on the calculations of brain tissue volume intended to be recruited by photo-stimulation, as per your experimental needs (Aravanis et al., 2007; Yizhar et al., 2011; Shin et al., 2016). Power outputs of up to 20 mW/mm^2^ are safe to use in neurons *in vivo* (Gradinaru et al., 2010; Fenno et al., 2011). Conversely, even sub-mW light intensities are sufficient to elicit optogenetics effects, although the induced voltage changes from the resting membrane potential will be understandably smaller (Madisen et al., 2012; Vazquez et al., 2013; Honjoh et al., 2014).

**FIGURE 6:**
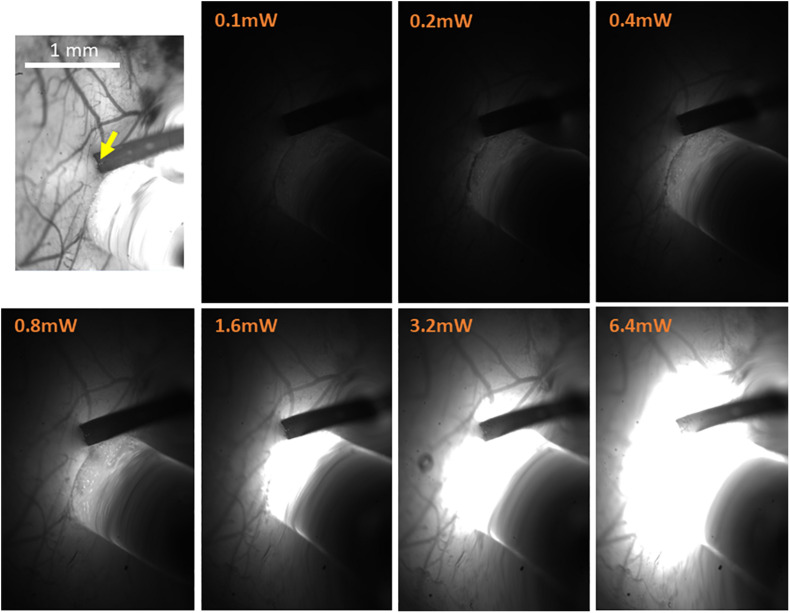
High-resolution imaging of the spatial extent of the optic fiber photostimulation. The top-left panel presents the high-resolution reference image taken under illumination wavelength of 530 nm, just prior to the electrophysiology recordings. It features the pial vessel topography and the electrode insertion site (yellow arrow). The scale bar represents 1 mm. The next seven panels show the optic fiber illumination of the region of interest at the indicated output power of amber light (wavelengths distribution centered on 590 nm; used with eNpHR3.0), taken in otherwise complete darkness and with identical imaging parameters.

While monitoring with the high-magnification lens attached to the OI-IS camera, slowly insert the electrode down to the desired cortical depth. This can be estimated by the number of contacts that remain visible above the cortical surface, and by taking into account the geometry of the probe, such as the arrangement of contacts and the distance between them (Figures 3, 6). Wait 5 min for the brain tissue to settle.

(6.7) Record the responses to the planned combinations of sensory stimulations and/or LED optogenetics photostimulation. We use the same experimental paradigm as in steps 3.1.2 and 4.6, except that we turn on the optogenetics photostimulation 2.25 s after the first sensory stimulation, and turn it off 2 s later. Applying the calculations from step 6.6, we use an exponential series of eight LED power outputs, from 0.1 to 12.8 mW, as measured at the tip of the optic fiber. Typically, this encompasses the full range of optogenetics effects, as 0.1 mW elicits negligible effects, whereas 12.8 mW virtually saturates the system. As a control, photo-stimulation in opsin-negative mice, whether wild-types injected with a Cre-dependent viral vector or mice expressing local Cre recombinase injected with a virus containing no opsin genome, should produce no observable optogenetics effects (Honjoh et al., 2014).

### (7) Post-experiment Histology Evaluation

**FIGURE 7:**
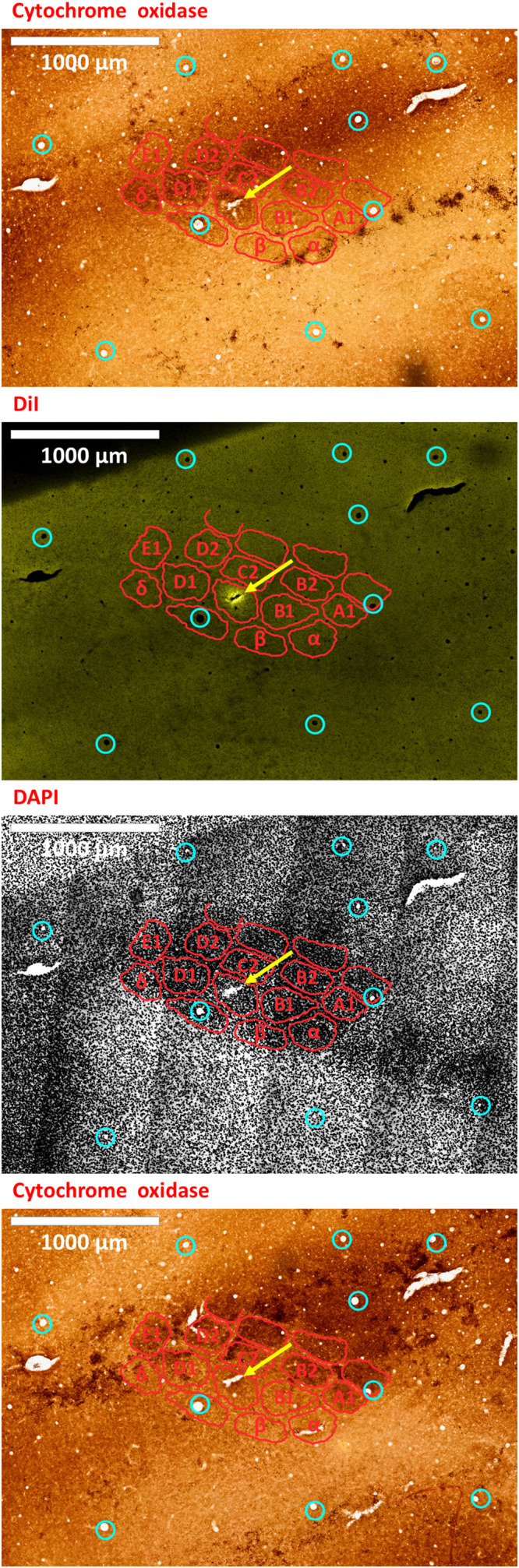
An electrode insertion into a pre-defined barrel is verified by DiI fluorescence co-localized with DAPI and cytochrome oxidase staining of the flattened cortex. Post-experiment histology was performed on the flattened cortex of the mouse’s right hemisphere, from which we present three consecutive slices. In all panels, red perimeters delineate the major barrels, as identified by cytochrome oxidase and DAPI, and yellow arrows point to the insertion within the targeted barrel. Penetrating blood vessels (marked with cyan circles) were used to optimize the finescale registration of the three adjacent slices using translation and rotation (see Shmuel et al., 2005). The top-most and bottom-most panels present brightfield images of cytochrome oxidase staining of the first and third slices, respectively, showing the targeted C1 barrel. The second and third panels represent the same second slice imaged using different filters; therefore, corresponding pixels are perfectly co-aligned between them. The second panel shows the electrode insertion site, imaged using a TRITC filter at 580 nm. The recording electrode was coated with DiI prior to insertion; thus, the DiI fluorescence image identifies the insertion site (yellow arrow). Twelve penetrating blood vessels (marked with cyan circles) were used to optimize the registration of consecutive slices using translation and rotation (see Shmuel et al., 2005). The DiI mark of the insertion site in the second panel shows that the insertion was inside the targeted C1 barrel in the top and bottom panels. The third panel shows the DAPI counterstaining of cell bodies’ nuclei of the same slice, performed using a DAPI filter (distribution of wavelengths centered on 455 nm). A grayscale filter was applied to the DAPI image in order to improve visualization of the barrel field. The scale bar represents 1 mm.

**FIGURE 8:**
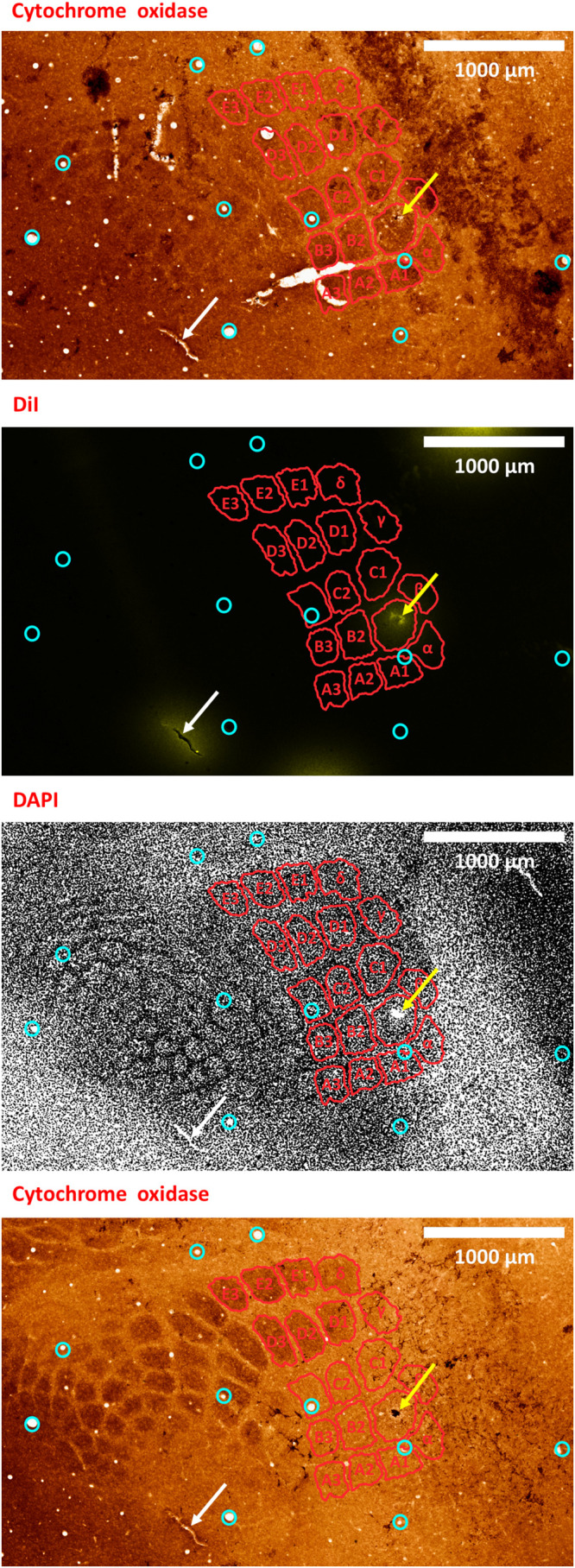
A second example of verifying an electrode insertion into a pre-defined barrel using tissue processing. Post experiment histology was performed on the flattened cortex of the mouse right hemisphere, from which we present three consecutive slices. In all panels, red perimeters delineate the major barrels identified in the second panel, as localized using both cytochrome oxidase and DAPI, yellow arrows point to the insertion within the targeted barrel, and white arrows point to one of the four user-made DiI fiducial markers. The top-most and bottom-most panels present bright-field images of cytochrome oxidase staining of the first and third slices, respectively, showing the targeted B1 barrel. The second and third panels present the second slice imaged using different microscope filters; therefore, every pixel is perfectly co-localized between them. The second panel presents the DiI fluorescence imaging of the second slice. It shows the DiI fiducial markers as well as the recording site, using a TRITC filter with wavelength distribution centered at 580 nm. The third panel shows the DAPI counterstaining of cell bodies’ nuclei of this same slice, performed using a filter with wavelength distribution centered at 455 nm. A grayscale filter was applied to the DAPI image in order to improve visualization of the barrel field. Four user-made DiI fiducial markers were used for the initial co-registration of the slices. Ten penetrating blood vessels (cyan circles) were then used to optimize the fine-scale registration of the consecutive slices. The DiI-marked electrode insertion site (yellow arrow) is inside the pre-selected B1 barrel. The scale bar represents 1 mm.

(7.1) At the end of the recording experiment, euthanize and perfuse the animal according to your institutional guidelines, using isotonic saline and 4% paraformaldehyde solution in phosphate buffered saline.
(7.2) Extract and fixate the brain. In order to confirm the location of the electrode, flatten the cortical hemisphere containing the ROI by removing the contralateral hemisphere if not needed (Strominger and Woolsey, 1987), gently scooping out the brainstem and sub-cortical parts, and placing a light flat weight made from a non-reactive material (we use an empty 15 mL glass Erlenmeyer flask), on top of the cortex, which will then be submerged in fixative.
(7.3) When fixation is complete, perform your histology protocol to obtain slices parallel to the cortical surface. Frozen fixed mouse brain blocks are sectioned to obtain 30 micron-thick slices using a cryostat (Leica Biosystems, Germany), although 40 microns is safer for fragile tissues. We use triple fluorescent slices [DiI and the opsins’ fluorescent tags, counterstained with 4’,6-diamidino-2-phenylindole (DAPI) to visualize cell bodies] in conjunction with interleaved slices stained with cytochrome oxidase to visualize S1BF barrels (Isett et al., 2018; Laboy-Juárez et al., 2019; Figures 7, 8). To verify opsin expression and tropism, a typical protocol involves successive steps in 0.1% Triton X-100 (MilliporeSigma, MA, United States) to permeabilize cell membranes; in normal donkey or horse serum (MilliporeSigma, MA, United States) step to minimize non-specific binding; in the primary antibody usually overnight; finally, in the secondary antibody with fluorescent tags, which comes from a different species than the primary (Dako, 2009; Yang et al., 2018).

## Results

Our first methodological objective is to inject an optogenetics virus in the mouse barrel field around a single barrel, to infect both this barrel as well as its immediate neighbors, while ensuring that the barrel itself is not damaged (Figures 3, 5). For guiding microinjections which are followed by the recovery of the animal, we propose to perform minimally invasive OI-IS through the thinned skull (Figures 2A, 3A, 4) and gently break the surface of the skull at the selected injection points.

Following an incubation period of 21–42 days (Zhang et al., 2010; Miyashita et al., 2013), we repeat the OI-IS in order to identify the target barrel and evaluate whether any injection-related damage could hamper its functionality (Figures 2B, 3B). This makes it possible to guide the insertion of an electrode within the barrel and optimize the positioning of the optic fiber attached to the LED or laser photo-stimulation. For this part of the experiment, we propose to perform a craniotomy, which makes it possible to obtain sharp images of the cortical surface, and to insert the electrode or multi-contact probe safely. In addition to guiding the insertion of the electrode to the pre-determined barrel, the user can use the OI system to estimate the spatial extent of the optogenetics photostimulation, by comparing the image obtained under the fiber-optic illumination to the pial vessels around the optic fiber (Figure 6).

In our experiments, the OI-IS hemodynamic responses obtained before the microinjections and following the incubation period were consistent: a single barrel’s localizations before and after the incubation period were always overlapping (Figures 2, 3). Thus, imaging post incubation is required for evaluating whether post-injection tissue damage interferes with the response of the module of interest or if the user needs to evaluate plasticity of the organization. Assuming that the virus on its own does not cause plasticity, the guidance of the neurophysiology can be based on the OI-IS results obtained before the microinjections and the topography of the cortical vessels (Figures 2, 3).

The expected outcome of the OI-IS is a well-delineated area of hemodynamic activation, verifying the original shape of – and centered on – the targeted structure. Ideally, two or more small structures such as barrels can thus be delineated and differentiated, with minimal overlap (Pouratian and Toga, 2002; Drew and Feldman, 2009; Figures 3, 4).

To validate our proposed method for OI-IS guided insertion of an electrode into a small cortical functional domain – a predetermined barrel, we performed histology of tissue slices cut tangential to the surface of the flattened brain. Barrels were stained with cytochrome oxidase, and cells were stained with the DAPI nuclear stain.

By default, the microinjection sites should not generate clearly visible long-lasting marks on the cortex. To mark the electrode insertion track, we dipped the electrode in DiI prior to insertion, in order to leave a fluorescent mark on a DAPI stained background, and to compare to the barrel field map obtained with cytochrome oxidase staining (Isett et al., 2018; Laboy-Juárez et al., 2019; Figures 7, 8). All our fluorescence histology slices were imaged at the appropriate filters for the three respective excitation wavelengths of DAPI, DiI and the opsin fluorescent tag. Therefore, each slice outputs three images that are perfectly co-aligned, with each pixel having a one-to-one spatial association in all three images. Thus, after performing alignment between the cytochrome oxidase and DAPI images, the DiI image needs only to be super-imposed on its matching DAPI image. Alignment of penetrating cortical blood vessels (Figures 7, 8) is used to optimize the fine-scale registration of two consecutive histology slices (Malach et al., 1993; Shmuel et al., 2005), even with different staining. Figures 7, 8 demonstrate that the insertion was into the center of the pre-determined barrel – validating the method we propose for guiding the microinjections and electrode insertion at high-precision.

## Discussion

Our proposed methodology aims to guide viral microinjections of viral vectors around – and electrode insertion into – a pre-defined small functional module. Given the delicate, long-term nature of optogenetics experiments as well as the efforts they require, it is important to have the viral microinjections and electrode insertions precisely in their intended locations.

### Optical-Imaging-Based Guidance of Optogenetics Viral Injections and Electrode Insertions

Optical Imaging of Intrinsic Signals is a widely used and easy to implement functional imaging technique with high spatial specificity and resolution (Grinvald et al., 1986). Relatively low-cost hardware is required for implementing OI-IS: a charge-coupled device or CMOS camera with a standard 50 mm – 60 mm lens, and an image acquisition system. The systems to generate the stimuli are required for the main experiment, independent of the OI-IS-based guidance that we propose. There are several facets of an optogenetics experiment that can be improved with this setup. These include the precise guidance of viral microinjections and the recording probe around or into a small functional module, and guiding the positioning of the optic fiber by estimating the region excited by the optogenetics illumination. By switching to a zoom lens, the experimenter can monitor the probe’s contacts at high-magnification, making it possible to control the depth of the electrode insertion.

While the current experiments have focused on barrels in mouse area S1BF, OI-IS can be used to localize several distinct cortical areas and modules of interest to deliver optogenetics viruses, photostimulate optogenetically, and record from multiple sites. Given its non-invasive nature, it would also be ideal for reading the long-term chronic-effects of optogenetics stimulation. Finally, OI-IS would be ideal for guiding optogenetics experiments in non-human primates and marmosets.

The main sources of error during OI-IS and their troubleshooting have been discussed in detail in the Results section. When used properly, this technique provides reliable, consistent identification of functional modules on the scale of hundreds of microns, to a degree of precision not attainable by using atlas coordinates and/or by trial-and-error of electrode insertion. We have demonstrated the accuracy of the OI-IS-guided insertions by post-experiment histology of the flattened brain. The DiI-marked electrode track is located inside the targeted barrel of interest, as identified by cytochrome oxidase and by counterstaining techniques such as DAPI. Additional immunohistochemistry options are available, such as using NeuN as the counterstain or using specific markers to identify the user’s particular target that would then be co-localized with the DiI from the recording site.

Whereas OI-IS requires craniotomy in large animals, it can be performed through the thinned skull in rodents. However, imaging through the skull blurs the images because of the light scattering caused by the bone. When imaging through the skull, a commonly observed issue is spatially blurred images of the pial vessels and hemodynamic response. The first step is to make sure that the lens is focused on the pial vessels. If the blurring persists, additional thinning of the skull may help. To overcome blurring when targeting small functional modules on the scale of hundreds of microns, imaging the cortex following craniotomy can be pursued (compare rows A and B in each of Figures 2, 3). Resecting the dura mater is required for imaging in large animals. In rodents – especially in mice – resecting the dura mater is not a condition for imaging; however, to obtain sharp images, it is recommended to resect the dura mater in rodents too. Therefore, the user can evaluate the tradeoff between the degrees of invasiveness against the spatial precision required for the guidance.

Another issue that we have commonly observed is that the activated region is larger than the corresponding anatomical structure (Figure 4), possibly because the stimulus may activate adjacent regions that are connected to the stimulated barrel (e.g., neighboring barrels) (Berwick et al., 2008; Drew and Feldman, 2009). To overcome this issue, the user can ensure a more balanced stimulus, such as reduced total duration, amplitude and/or frequency of whisker deflection (Berwick et al., 2008). Importantly, a hemodynamic response to stimulating a single module can overlap with neighboring modules. To address this issue, we recommend to image the responses to stimulation of the neighboring modules separately. Then, differential analysis of the different responses can be used to remove the common response and present the spatial contrast, as we as we demonstrate in Figure 4 (Bonhoeffer and Grinvald, 1993; Shmuel and Grinvald, 1996; Grinvald et al., 1999). Differential analysis eliminates the common response and enhances the visualization of the specific representation of the stimulus/module/barrel of interest (Figure 4).

Illuminating at an isosbestic wavelength of 550, 569, or 586 nm measures the total Hb content and, by extension, the CBV (Frostig et al., 1990). Functional imaging studies indicate that CBV responses co-localize faithfully to sites of increases in neural activity (Fukuda et al., 2006; Yao et al., 2017) whereas the patterns of changes in deoxygenated blood are most prominent in draining veins (Hess et al., 2000; Sheth et al., 2003, 2004; Lu et al., 2004; Hillman, 2014; Chaimow et al., 2018). Given the importance of spatial precision in identifying the pre-defined cortical column, we exclusively use green 530 nm illumination for both surface vasculature reference images and OI-IS. While 530 nm illumination shows a clear pattern of the pial vessels, it also provides the best contrast to noise (CNR) ratio. In other words, it gives a clear functional image in a short time frame. This feature is important for using OI-IS for guiding microinjections and insertion of electrodes to small functional modules, because the OI-IS stage has to be short. In addition, green illumination reflects changes in CBV, which show spatial specificity to the site of increased neuronal activity at a level comparable to that obtained by the OI-IS initial deep (Fukuda et al., 2006).

A critical aspect of OI-IS is the need of maintaining appropriate anesthesia, as this may influence both the neuronal and hemodynamic responses (Janssen et al., 2004; Masamoto and Kanno, 2012; Juavinett et al., 2017). It is critical for the quality of the experiment to avoid anesthetics that interfere with the cortical blood flow or with neurovascular coupling (Masamoto and Kanno, 2012). Isoflurane represents a non-optimal choice, as it depresses evoked responses and is a vasodilator at typical regimes (Iida et al., 1998; Bortel et al., 2020). In our mouse experiments, we use a combination of ketamine and xylazine or dexmedetomidine and isoflurane administered at low-percentage (Bortel et al., 2020).

### OI-IS Is Optimal for Guidance of Insertions Around and Into Fine-Scale Cortical Modules

Optical Imaging of Intrinsic Signals relies solely on intrinsic neurovascular elements and does not require adding an extrinsic indicator of neuronal activity (Grinvald et al., 1999). Thus, it requires no additional injections of viruses for the purpose of imaging (Seidemann et al., 2016), which may damage the cortical module of interest.

Membrane-bound dyes such as voltage-sensitive dyes (VSD) used *in vivo* report voltage changes in neurons at an excellent temporal and spatial resolution (Grinvald et al., 1988, 1999; Grinvald and Hildesheim, 2004; Devonshire et al., 2013). The VSD pharmacological and cytotoxic side effects have recently been alleviated to near-negligible levels, using newer generations of blue dyes and lower dye concentrations (Ross et al., 1977; Grinvald et al., 1988; Shoham et al., 1999; Grinvald and Hildesheim, 2004; Lippert et al., 2007; Grandy et al., 2012; Devonshire et al., 2013; Habib et al., 2013; Carter and Shieh, 2015). Compared to *in vivo* VSD imaging, OI-IS is an indirect indicator of neural activity. Nevertheless, while VSD imaging has undeniable advantages for imaging neuronal membrane potentials, OI-IS provides faster functional mapping in space because VSDs require 1–2 h to penetrate cortex and bind to the neurons’ membranes (Shoham et al., 1999; Lippert et al., 2007; Grandy et al., 2012; McVea et al., 2012). Obtaining the mapping from OI-IS faster than with VSD is important for OI-based guidance of viral microinjections, because it reduces the time under anesthesia in recovery experiments. Similarly, in acute experiments, OI-IS makes it possible to start the neurophysiological recordings earlier than VSD does, thus reducing effects of accumulated anesthesia during the recordings.

In addition, OI-IS is minimally invasive when performed through a thinned skull, whereas VSD imaging requires craniotomy. Although VSD imaging can be successfully combined with optogenetics (Willadt et al., 2014), it requires a judicious choice of dyes and opsins and a more advanced photostimulation/imaging setup (Vogt et al., 2011; Willadt et al., 2014). We posit that for guiding insertions of microinjection pipettes and/or electrodes into fine-scale functional modules, OI-IS is superior to VSD imaging.

### Consideration of Selecting the Viral Vector, Serotype, and Promoter for Applying Optogenetics in Fine-Scale Cortical Modules

For combining OI-IS with optogenetics, it is important to consider the bands of wavelengths for the OI-IS illumination and for exciting the optogenetic opsin. If these distributions overlap considerably, the OI-IS illumination will excite the optogentic opsin, which will, in turn, manipulate the neuronal activity. This is especially important if the OI-IS serves as a readout to quantify the effect of the optogenetic manipulation. However, to prevent undesired effects, it is important to consider the distributions of wavelengths also for using OI-IS to guide the insertion of probes into brains that already carry the optogenetic opsin. Here we imaged relative changes in total hemoglobin using a narrow band of wavelengths centered on 530 nm. The optogenetics opsin we used is ChR2, whose maximum sensitivity is at 466 nm (Nagel et al., 2003; Zhang et al., 2010; Yizhar et al., 2011). At 530 nm, ChR2’s sensitivity drops to 21% of the maximum sensitivity. Given that the power used for OI-IS illumination is lower than that used for optogenetics, we expect that the effect of the OI-IS illumination on the neuronal activity through excitation of the optogenetics opsin is diminished.

In animal models, opsins are commonly introduced into neurons via viral microinjections (Bernstein and Boyden, 2011; Fenno et al., 2011; Yizhar et al., 2011). Recently, adeno-associated viruses (AAV) have become favored because of their low immunogenicity, good production titer, and expression efficiency, but especially because they can be manipulated in Bio-Safety Level 1 conditions (Zhang et al., 2010; Fenno et al., 2011; Thompson and Towne, 2018; Addgene, 2020). The user can control the degree of specificity of the optogenetics manipulation by using different viral characteristics and microinjection parameters (Gradinaru et al., 2010; Chernov et al., 2018). For example, some viral capsids are taken up into cells faster than others, thus modulating the volume of infection; the promoter can allow expression in an exclusive type of cells or tissue, which is termed tropism; the serotype, viral type, and genome can influence the levels of expression, antero- or retrograde transport, and *trans*-synaptic infection (Cearley and Wolfe, 2006; Cohen et al., 2011; Fenno et al., 2011; Yizhar et al., 2011; Urban and Rossier, 2012; Aschauer et al., 2013; Scheyltjens et al., 2015; Watakabe et al., 2015; Thompson and Towne, 2018; Yizhar and Adamantidis, 2018).

**FIGURE 9:**
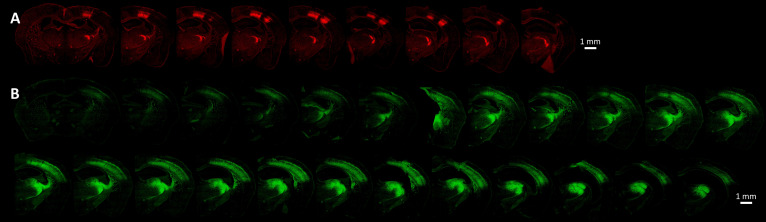
Cortical opsin expression is significantly dependent on viral and microinjection characteristics such as the promoter-serotype combination. **(A)** Presents the opsin expression from the virus AAV2/8-CAG-Flex-ChR2-tdTomato (titer of 1e13 GC/ml). The coronal sections in the bottom panel show the spread of the virus AAV2/5-EF1α-DIO-ChR2-EYFP (titer of 5e12 GC/ml). Both viruses were injected at two locations determined by OI-IS, with each location receiving two injections of 150 nL at a rate of 20 nL per minute. In each of these cases, injections were made at two cortical depths, 700 microns, and 900 microns. The imaged slices were 30 micron-thick. The images were taken at 10x magnification by an Olympus VS120 slide scanner, using the orange (TRITC, 580 nm) and green (FITC, 518 nm) channels, respectively.

The viral and microinjection characteristics influence directly the opsin expression, but also the experimental approach. The promoter-serotype combination is the most significant intrinsic factor determining the viral infection spread and pattern, although some variations may occur, especially at extreme titers (Nathanson et al., 2009; Yizhar et al., 2011; Aschauer et al., 2013; Scheyltjens et al., 2015). For example, chicken β-actin (CBA), its derivative called CAG, and human cytomegalovirus (CMV) are generally considered strong transcription promoters (Powell et al., 2015), while CaMKIIα 0.4 constructs specifically infect more than 90% excitatory neurons (Yizhar et al., 2011; Scheyltjens et al., 2015). The new hybrid vector AAV-DJ combines elements from eight different serotypes to achieve high transduction efficiency (Grimm et al., 2008; Thompson and Towne, 2018). The cortical spread of various recombinant, hybrid AAV serotypes using either the CMV or the CaMKIIα promoters, shows an increasing serotype efficacy of 2/1 << 2/7 ∼ 2/8 ∼ 2/9 < 2/5 (in this terminology, the AAV2 inverted terminal repeat has been cross-packaged in the capsid from the second numbered serotype, Choi et al., 2005), based on mean expression spread from the injection site (Scheyltjens et al., 2015). Other researchers have found serotype 2/8 to spread less than 2/9 (Cearley and Wolfe, 2006; Aschauer et al., 2013; Thompson and Towne, 2018), but since they share axonal transport mechanisms (Castle et al., 2014), a possible explanation would be that the uptake of 2/8 into neurons is faster and thus the virus has less time to spread, possibly due to improved uptake through the plasma membrane. Therefore, if the experiment requires a smaller confined area of opsin expression, then a good option is to inject AAV2/8 with the CMV promoter, as long as the microinjection sites can be positioned less than 1 mm apart from each other and from the center of the cortical module of interest (Scheyltjens et al., 2015). If the region of interest (ROI) is widespread, forcing the microinjection sites to be too numerous or far away from each other, then 2/5 or 2/9 can be used instead (Scheyltjens et al., 2015; Thompson and Towne, 2018). See an empirical comparison of a narrow spreading expression of AAV2/8-CAG versus a far-spreading AAV2/5-EF1α in the top and bottom panels of Figure 9, respectively. Since cell tropism and infection efficiency may vary with the location and type of tissue being targeted (Burger et al., 2004), the best practice is to test several viral vectors to compare the resulting opsin expressions empirically.

## Conclusion

Pursuing optogenetic microinjections or recording neurophysiology from inside a predetermined fine-scale cortical module requires a careful consideration of the experimental parameters, such as viral serotypes and promoter. More importantly, it also requires to precisely map these modules *in vivo*. The OI-IS-based guidance methodology described in this manuscript makes it possible to insert micropipettes for viral microinjection and neurophysiology electrodes quickly and accurately to their pre-determined functional module. It allows sub-millimeter spatial resolution and minimal overlap of activated modules. It also features a low degree of invasiveness; thus, it is safe for use in long-duration protocols such as microinjections of optogenetic viral vectors in a recovery surgery, followed by a period of several weeks allowing opsin expression, then performing the readout and/or behavioral measurements.

## Data Availability Statement

The data supporting the conclusions of this article will be made available upon a request sent by email to the corresponding author. See https://www.mcgill.ca/neuro/amir-shmuel-phd.

## Ethics Statement

This animal study was reviewed and approved by the Animal Care Committee of the Montreal Neurological Institute, McGill University.

## Author Contributions

VMM designed the study, acquired and analyzed the data, and wrote the manuscript. AS initiated and designed the study, oversaw the data acquisition and analysis, wrote part of the code for the OI-IS data analysis, and wrote the manuscript. Both authors contributed to the article and approved the submitted version.

## Conflict of Interest

The authors declare that the research was conducted in the absence of any commercial or financial relationships that could be construed as a potential conflict of interest.
